# Psychometric properties of Patient Reported Outcome Measures (PROMs) in patients diagnosed with Acute Respiratory Distress Syndrome (ARDS)

**DOI:** 10.1186/s12955-016-0417-7

**Published:** 2016-01-27

**Authors:** Hiral Anil Shah, Melina Dritsaki, Joshua Pink, Stavros Petrou

**Affiliations:** Public Health Foundation of India, Delhi NCR, India; Warwick Clinical Trials Unit, Division of Health Sciences, Warwick Medical School, University of Warwick, Coventry, CV4 7AL UK; Oxford Clinical Trials Research Unit, Nuffield Department of Orthopeadics, Rheumatology and Musculosceletal Sciences, University of Oxford, Oxford, OX3 7HE UK

## Abstract

**Background:**

The aim of this study was to assess the psychometric properties of the EQ-5D-3 L, the SF-12 v2 and its preference based derivative the SF-6D, and the St Georges Respiratory Questionnaire (SGRQ), in patients diagnosed with Acute Respiratory Distress Syndrome (ARDS).

**Methods:**

Data from the Oscillation in ARDS (OSCAR) randomised unblinded clinical trial of 795 patients diagnosed with ARDS provided the foundation of this secondary psychometric analysis. The three source patient reported outcome measures (PROMs) (EQ-5D-3 L, SF-12 and SGRQ) were collected at both 6 and 12 months post randomisation. All measures were tested for acceptability, reliability, internal consistency, validity and responsiveness. Data from responders at 6 months was used to test for acceptability, reliability, known groups validity and internal responsiveness. Data from patients who responded at both 6 and 12 months was used to test for convergent validity and external responsiveness.

**Results:**

Rates of response at both 6 and 12 months post randomisation were 89.88 % for the EQ-5D-3 L, 77.38 % for the SF-6D, 71.43 % for both the physical and mental components of the SF-12 and 38.10 % for the SGRQ. All measures had a Cronbach’s Alpha statistic higher than 0.7. For known group’s validity, there was no difference in mean summary or utility scores between known groups for all PROMs with minimal effect sizes. All three source measures showed strong convergent and discriminant validity. There was consistent evidence that the SF-6D is an empirically valid and efficient alternative to the EQ-5D-3 L. The EQ-5D-3 L and SGRQ were more responsive compared to the SF-12 and SF-6D with the EQ-5D-3 L generating greater effect sizes than the SGRQ.

**Conclusion:**

The PROMs explored in this study displayed varying psychometric properties in the context of ARDS. Further research should focus on shortening the SGRQ whilst still maintaining its psychometric properties and mapping between the SGRQ and preference-based measures for future application within economic evaluations of respiratory focused interventions. The selection ofa preferred PROM for evaluative studies within the ARDS context should ultimately depend on the relative importance placed on individual psychometric properties and the importance placed on generation of health utilities for economic evaluation purposes.

## Background

Acute Respiratory Distress Syndrome (ARDS) is a severe life threatening condition, which develops if the lungs become severely inflamed due to an infection or injury. Although there is a low incidence (approximately 78–280 cases per million population), ARDS is associated with a high mortality rate of 40 % or greater [1–9]. It is estimated that due to long intensive care unit and hospital stays, the cost of every saved life from ARDS is approximately £43,000 (2010 prices) [10, 11]. Patients who survive ARDS tend to have a high number of comorbidities and a poor health-related quality of life (HRQoL) with 35 % unable to return to work 24 months after hospital discharge [12, 13]. Health care costs tend to increase after surviving ARDS due to the need for hospital readmission and inpatient rehabilitation [14].

Patient reported outcomes (PROs) such as symptoms and health utilities can be measured through self-reported questionnaires of health status or HRQoL, which are completed by patients at different time points and are otherwise known as patient reported outcome measures (PROMs). PROMS can be used to compare patients’ self-reported health status or HRQoL at two separate points in time, allowing analyses of the change in health status or HRQoL with respect to an intervention [15, 16]. The inclusion or use of poorly designed or inadequately targeted PROMs in a study that has not considered their psychometric properties can have adverse consequences. These include an additional burden to the patient, an increase in study costs and ethical concerns surrounding patients having to complete measures that are incapable of capturing the patient’s perspective [17]. This could also lead to missing data, unreliable information and biased results. These consequences should therefore be avoided with further research and evidence of the psychometric properties of PROMs in particular populations.

The OSCAR (Oscillation in ARDS) study was conducted to assess the effectiveness and cost-effectiveness of High Frequency Oscillatory Ventilation (HFOV) against conventional artificial ventilation for adults with ARDS. The OSCAR study included the EQ-5D-3 L, SF-12 and the St Georges Respiratory Questionnaire (SGQ) in patients diagnosed with moderate to severe ARDS.

There is limited evidence regarding the psychometric properties of PROMs used in critical care and no evidence in patients with ARDS [18–21]. Menn et al [18] found that in patients with severe Chronic Obstructive Pulmonary Disorder (COPD) hospitalised for exacerbations, the EQ-5D-3 L appeared to be a suitable measure of HRQoL, whereas the SF-12 appeared to be less suitable for a self-assessment due to the high proportion of missing values. Additionally, the psychometric properties of the SGRQ were satisfactory in this population group, although there was a recognition that no utility values (preference based outcomes) could be derived using this PROM [18]. Therefore, considering that there has been limited information on the properties of alternative PROMS in critical care, and no previous assessments in the context of ARDS, the objective of this study was to assess the psychometric properties of the EQ-5D-3 L, the SF-12 and its preference based derivative the SF-6D, and the SGRQ, in patients diagnosed with ARDS.

## Methods

### Study population

The data used in this study was derived from the OSCAR trial, which was a randomised unblinded controlled trial with a prospective cost utility analysis [22, 23]. Further details regarding the OSCAR trial are available in the published literature [22–24].

Patients did not complete any PROMs at baseline as they were intubated at that stage. Patients were followed up at 6 and 12 months after randomisation using self-complete postal questionnaires, which contained the EQ-5D, SF-12 v2 (hereafter SF-12 for brevity) and the SGRQ [24].

### Patient reported outcome measures

The EQ-5D-3 L is a generic preference based questionnaire, which asks patients about their health status on the day they complete the questionnaire. The EQ-5D-3 L has five separate dimensions: mobility, self care, usual activities, pain/discomfort and anxiety/depression. Each of these dimensions has three response levels (no problems, some or moderate problems and severe or extreme problems) [25, 26]. Therefore, there are a possible 243 (3^5^) health states that can be generated from the EQ-5D-3 L descriptive system. The EQ-5D is generally valued using a time-trade method. For the purposes of this study, we applied the York A1 (Dolan) tariff set derived from a survey of the UK general population (*n* = 3337), which used the time trade-off valuation method to estimate utility scores for a subset of 45 EQ-5D health states, with the remainder of the EQ-5D health states subsequently valued through the estimation of a multivariate model [27]. Resulting utility scores range from -0.59 to 1.0, with 0 representing death and 1.0 representing full health; values below 0 indicate health states worse than death. The EQ-5D-3 L visual analogue scale (VAS) was not used within the OSCAR trial.

The SF-12 is a generic non-preference based PROM which contains 12 questions selected from a parent PROM called the SF-36. The SF-12’s questions are designed to provide patients or individuals with the opportunity to recall their health status retrospectively over a 4 week period. This questionnaire has eight separate dimensions [28, 29]. The SF-12 measures various aspects of physical and mental health from which physical (PCS) and mental (MCS) component scores can be calculated [25]. Whilst such scores provide a method for analysing the effectiveness of interventions, they have only a limited application in economic evaluations because they are not based on population preferences. Hence a six dimension health state classification can also be constructed from the SF-12 called the SF-6D. The SF-6D is a preference based measure that can generate 18,000 health states which can be converted into utility values (ranging from 0.345 to 1.0) using a set of preference weights obtained from the UK general population and valued using the standard gamble valuation technique [30].

The St Georges Respiratory Questionnaire (SGRQ) is a 50 item condition specific non-preference based PROM developed to measure health status in patients with diseases of airways obstruction. Three component scores for the dimensions of symptoms, activity and impact on daily life can be calculated and a total score can also be calculated from the SGRQ. The symptoms component score is concerned with the effects of respiratory symptoms, their frequency and severity. The activity component scores is concerned with activities that cause or are limited by breathlessness. The impact score is concerned with social functioning and psychological disturbances resulting from airways disease. The total score summarises the impact of the disease on overall health status. Scores can range from 0–100 with a higher score representing a lower respiratory health [31].

### Statistical analysis

Baseline characteristics and descriptive characteristics were computed. The difference between scores, characteristics and utility values were tested using the unpaired t-test for continuous variables and chi-squared test for categorical variables and presented within tables. Missing data was excluded from all statistical analyses. As there were no significant effects from the trial intervention, patients within the OSCAR trial were pooled for these secondary analyses regardless of trial allocation [24]. Data from responders at 6 months was used to test for acceptability, reliability, known groups validity and internal responsiveness. Data from patients who responded at both 6 and 12 months was used to test for convergent validity and external responsiveness.

The psychometric properties of each study PROM, including its acceptability, internal consistency, reliability, validity and responsiveness was assessed. This was based on the COSMIN taxonomy [32] and a previously published checklist of assessment criteria for PROMs [33].

### Acceptability

The acceptability of the different study PROMs was measured using completion rates of the different instruments at 6 months post randomisation [25, 34].

### Reliability

Internal consistency, which is a measure of reliability, assesses whether several items that propose to measure the same general dimensions produce similar results. The internal consistency of each PROM was measured by calculating its Cronbach’s alpha statistic. A commonly accepted categorisation for internal consistency has been to consider scores between 0.7 and 0.8 to be acceptable, 0.8 and 0.9 to represent good reliability and 0.9 and higher to represent excellent reliability [33].

### Construct validity

There are three common approaches to measuring construct validity: known groups, convergent and discriminant. Known groups’ comparisons were conducted using groups categorised by the following baseline indicators: PaO_2_/FiO_2_ ratio [35] and APACHE II [36] scores. Using the 2012 “Berlin criteria” produced by the European Society of Intensive Care Medicine, OSCAR patients were classified into moderate or severe ARDS based on their decreased PaO_2_/FiO_2_ ratio. If the ratio was less than 13.3kPa then the patient was classified as a severe ARDS patient and if it was greater than 13.3kPa then the patient was classified as a moderate ARDS patient [22, 23, 35, 37]. Within the OSCAR study, the APACHE II score was used to compute the risk of dying and thus the severity of illness. An APACHE II score higher than 26 indicated a less than 50 % chance of survival and an APACHE II score lower than or equal to 26 indicated a more than 50 % chance of survival [22].

This analysis was conducted using independent t-tests for differences at 6 months for all study PROMs. The magnitude of the difference was estimated by calculating the Cohen’s D effect size. A standard classification of Cohen’s D effect sizes regards a value of 0.20 as a small response, 0.50 as a moderate response and 0.80 or greater as a large response [38].

Convergent and discriminant validity is the extent to which PROMs with overlapping dimensions and constructs may be similar or different. It is expected that similar constructs between PROMs (e.g. pain in the EQ-5D-3 L and pain in the SF-12) should correlate [25]. The Pearson’s R correlation coefficients were calculated between summary scores and utility values to test for convergent and discriminant validity. Dimensions and domains were then correlated amongst the EQ-5D-3 L, SF-12, SF-6D and SGRQ. Spearman ranks correlation was used to assess whether there was a relationship for all dimensions to ascertain convergent or discriminant validity with the assumption that similar dimensions in different measures should correlate more than different dimensions within the same measure. A higher correlation between a generic source PROM and the SGRQ can be considered as evidence of a greater degree of construct overlap between the generic measure and the SGRQ [25].

### Empirical validity

Empirical validity has been defined as whether a preference-based measure generates utility scores that reflect people’s preferences whether revealed stated or hypothesised [39]. Empirical validity was tested using the relative efficiency (RE) statistic to detect differences in an external measure of health status. This test was only conducted on the EQ-5D-3 L and the SF-6D (our two preference-based measures). This test could not be conducted on the SGRQ as it is a condition specific PROM that is not preference based. Additionally, this test was distinguished from the other tests of validity that are applied to all the PROMs. In order to calculate the RE statistic, all responders at 6 months were dichotomised using an external measure of current general health (derived from Question 1 of the SGRQ) and current respiratory health (derived from the SGRQ Total Score). Current general health was dichotomised as very good or good versus fair, poor or very poor [39]. The dichotomisation for current reported respiratory health was an SGRQ total score of less than or equal to 40 (considered to be very good or good respiratory health) versus an SGRQ total score of more than 40 (considered to be fair, poor or very poor respiratory health). Previous research into the validity of the SGRQ in COPD patients used a threshold of 33 for the SGRQ total score to identify COPD; however, considering the severity of ARDS, the authors felt that a threshold of 33 would be too low to dichotomise an ARDS population [21, 40].

### Responsiveness

Responsiveness was categorised into internal and external responsiveness. Responsiveness can be assessed by examining floor and ceiling effects of the measure to determine the extent to which a person can move on the scale if their HRQoL changes over time [41]. Further testing to determine internal and external responsiveness could not be conducted between baseline and follow up periods. We did however test for external responsiveness of the EQ-5D-3 L, SF-12, SF-6D and SGRQ between 6 and 12 months. Here an external reference measure was provided by the SF-12 Question 1, which asked about current general health with possible responses of very good, good, fair, poor or very poor at both 6 and 12 months used to categorise participants. This reference measure was chosen as it was not used in the calculation of any utility or summary scores. This objective was to test whether the changes registered by a measure over time resemble those expected based on an external measure of health [25, 42]. Mean differences calculated by paired t-tests and standardised response means were calculated to ascertain changes in the outcome measures for patients in the self-reported (current general health) groups. A larger difference between groups indicated a more responsiveness measure [25].

## Results

A total of 795 patients were randomised within the OSCAR study of whom 168 were complete study responders, meaning all three source PROMs (EQ-5D-3 L, SF-12 & SGRQ) were completed and returned at both 6 months and 12 months post randomisation. The baseline characteristics of the study population are shown in Table 1. There was no difference between patients who responded at both 6 and 12 months, incomplete responders and patients who did not complete any questionnaires at either 6 or 12 months follow up in terms of sex or ARDS type. However, there was a significant difference in average age between all three groups (*P* < 0.001). Incomplete responders tended to be younger than both patients who responded to questionnaires at both follow up points and also patients who did not complete any questionnaires at either follow up point (*P* < 0.05). There was also a difference in average weight between patients who responded at both follow ups and patients who did not complete any questionnaires at either follow up point (*P* < 0.001).

**Table 1:** Baseline characteristics of the OSCAR study population

	Whole population (*n* = 795)	Responders at both 6 and 12 months (*n* = 168)	Incomplete responders (*n* = 101)	Patients without any PROMs data (*n* = 526)
Mean Age (SD)	54.90 (16.76)	54.82 (15.10)	47.39 (16.08)	56.37 (17.03)
Mean Weight (SD)	79.7 (21.35)	83.18 (19.49)	80.05 (25.76)	78.53 (20.90)
Male (%)	495 (62.3 %)	114 (67.9 %)	62 (61.4 %)	319 (60.6 %)
Female (%)	300 (37.7 %)	54 (32.1 %)	39 (38.6 %)	207 (39.4 %)
Moderate ARDS (%)	478 (59.9 %)	107 (63.7 %)	61 (60.4 %)	308 (58.6 %)
Severe ARDS (%)	319 (40.1 %)	61 (36.3 %)	40 (39.6 %)	218 (41.4 %)

SD denotes standard deviation. “Patients without any PROMs data” includes patients who died before follow up and survivors who didn’t respond to either follow up questionnaire

The unit for age was in years. The unit for weight was in kilograms. Male, Female, Moderate & Severe ARDS are shown as absolute numbers with the proportion of each gender or ARDS type shown in brackets

### Descriptive statistics

Descriptive statistics were calculated for each outcome measure and are shown in Table 2. Here the EQ-5D-3 L produced lower utility values compared to the SF-6D at 6 months post randomisation. Table 2 also summarizes levels of floor and ceiling effects for all PROMs. The EQ-5D-3 L showed evidence of ceiling effects. There was no evidence of a floor or ceiling effect for the SF-6D or the SF-12. A ceiling effect was seen for all the summary scores of the SGRQ and a floor effect for the SGRQ activity score.

**Table 2:** Descriptive statistics for each patient reported outcome measure used in the 6 month follow up post randomisation in the OSCAR trial

	Range	N	Mean	SD	Response rates	Floor effects	Ceiling effects
EQ-5D-3 L (Utility)	−0.594 to 1	234	0.60	0.36	95.29 %	0.00 %	19.73 %
SF-6D (Utility)	0.345 to 1	218	0.66	0.16	93.16 %	0.92 %	0.92 %
SF-12 PCS	0 to 100	209	36.88	12.32	89.32 %	0.00 %	0.00 %
SF-12 MCS	0 to 100	209	43.43	13.30	89.32 %	0.00 %	0.00 %
SGRQ Symptoms Score	0 to 100	221	33.71	27.89	94.44 %	1.36 %	1.36 %
SGRQ Activity Score	0 to 100	193	46.04	31.80	82.47 %	7.77 %	7.77 %
SGRQ Impact Score	0 to 100	194	22.13	22.42	82.90 %	0.00 %	0.00 %
SGRQ Total Score	0 to 100	175	32.08	24.18	74.79 %	0.00 %	0.00 %

Scores or Utility Values of participants which could not be calculated due to missing items were not included within the analysis

N stands for the sample size. SD denotes the standard deviation. CI is abbreviated for confidence intervals

Floor effects is the proportion of patients in the lowest health state possible and ceiling effects is the proportion of patients in the highest health state poss

EQ-5D-3 L is anchored at -0.594 for worst possible health and 1 for best possible health

SF-6D is anchored at 0.345 for worst possible health and 1 for best possible health

SF-12 PCS & MCS scores are anchored at 0 for worst possible health and 1 for best possible health

SGRQ scores are anchored at 100 for worst possible health and 0 for best possible health

The EQ-5D-3 L and SF-12 was assessed in terms of complete responses for all questions

The SF-6D was regarded complete if there was sufficient items to calculate a score

The SGRQ was assessed by calculating if more than 24 % of the items were missing or not answered, then a total score could not be calculated

### Acceptability

Table 2 also shows the response rates for each PROM at 6 month follow up post randomisation. Response rates varied between 95.29 and 74.79 % at 6 months. The EQ-5D-3 L had a very high response rate, whilst the SF-6D had response rates that were marginally greater than the SF-12 components (PCS and MCS scores). The SGRQ had a wide range of response rates with the symptoms score generating the greatest response compared to the activity, impact and total scores. The SGRQ total score was also found to have had the lowest response rate.

### Reliability

All three source PROMs generated Cronbachs alpha statistics greater than 0.7, which was deemed acceptable for research purposes [43]. Cronbach's alpha scores were found to be 0.732, 0.880 and 0.963 for the EQ-5D-3 L, SF-12 and the SGRQ respectively.

### Validity

#### Construct validity

The results for the tests of known groups’ validity are summarized in Tables 3 and 4. The known group validity test had to be conducted at 6 months due to the absence of baseline values at randomisation. The difference in the scores for all measures specified by baseline APACHE II scores and PaO_2_/FiO_2_ ratios indicated that there was no difference between the known groups for all PROMs with minimal effect sizes.

**Table 3:** Known groups validity – APACHE II scores

	APACHE score < =26 mean (SD)	APACHE score < =26 n	APACHE score >26 Mean (SD)	APACHE score >26 n	Difference	*P* value	Confidence intervals		Cohen’s D
EQ-5D-3 L (Utility)	0.61 (0.35)	195	0.47 (0.40)	28	0.14	0.058	−0.05	0.28	0.38
SF-6D (Utility)	0.64 (0.15)	191	0.62 (0.16)	27	0.05	0.155	−0.02	0.12	0.29
SF-12 PCS	36.90 (12.45)	185	36.70 (11.51)	24	0.20	0.942	−5.09	5.48	0.02
SF-12 MCS	45.99 (13.24)	185	41.12 (13.21)	24	4.87	0.001	−0.79	10.55	0.37
SGRQ Symptoms Score	33.04 (28.00)	194	38.57 (27.05)	27	−5.54	0.334	−16.83	5.75	0.20
SGRQ Activity Score	45.91 (31.26)	169	48.63 (36.07)	24	2.72	0.696	−16.44	10.99	0.09
SGRQ Impact Score	22.08 (22.46)	169	22.49 (22.65)	25	0.41	0.932	−9.91	9.09	0.02
SGRQ Total Score	31.95 (23.97)	154	33.04 (26.29)	21	1.09	0.847	−12.22	10.05	0.04

SD denotes standard deviation

**Table 4:** Known groups validity – PaO2/FiO2 ratio

	Moderate ARDS mean (SD)	Moderate ARDS n	Severe ARDS mean (SD)	Severe ARDS n	Difference	*P* value	Confidence interval		Cohen’s D
EQ-5D-3 L (Utility)	0.60 (0.39)	141	0.58 (0.31)	82	0.02	0.749	−0.08	0.16	0.04
SF-6D (Utility)	0.66 (0.16)	138	0.65 (0.14)	80	0.01	0.586	−0.03	0.06	0.08
SF-12 PCS	37.60 (12.49)	131	35.66 (12.02)	78	1.93	0.274	−1.54	5.41	0.16
SF-12 MCS	46.04 (12.80)	131	44.41 (14.12)	78	1.64	0.391	−2.11	5.39	0.12
SGRQ Symptoms Score	31.73 (27.31)	140	37.15 (28.72)	81	−5.42	0.164	−13.08	2.24	−0.19
SGRQ Activity Score	43.59 (31.69)	120	50.62 (31.71)	73	−7.03	0.137	−16.31	2.25	−0.22
SGRQ Impact Score	20.57 (22.14)	120	24.65 (22.80)	74	−4.08	0.219	−10.61	2.44	−0.18
SGRQ Total Score	37.14 (28.72)	108	35.18 (23.98)	67	−5.03	0.182	−12.44	2.38	−0.21

#### Convergent and discriminant validity

Table 5 shows Pearsons R correlation coefficients between various summary and outcome measures. The majority of summary and outcome measures correlated however, the PCS and MCS of the SF-12 did not significantly correlate. The EQ-5D-3 L utility scores correlated more strongly with the SF-6D utility score. The EQ-5D-3 L utility scores also correlated moderately with the SF-12 PCS component score [44]. There was a strong correlation between component SGRQ scores (symptom, activity, impact and total) at the statistically significant 5 % level. Lastly, there was weak correlation between SGRQ scores and the EQ-5D-3 L, SF-6D and both SF-12 components (PCS and MCS) [44].

**Table 5:** Convergent & discriminant validity - Pearson’s R correlation

	EQ-5D-3 L (Utility)	SF-6D (Utility)	SF-12 PCS (Utility)	SF-12 MCS (Utility)	SGRQ Symptoms score (Utility)	SGRQ Activity score (Utility)	SGRQ Impact score (Utility)	SGRQ Total score (Utility)
EQ-5D-3 L (Utility)	1.00							
SF-6D (Utility)	0.77	1.00						
SF-12 PCS	0.60	0.62	1.00					
SF-12 MCS	0.49	0.67	−0.07	1.00				
SGRQ Symptoms Score	−0.35	−0.43	−0.30	−0.39	1.00			
SGRQ Activity Score	−0.38	−0.44	−0.46	−0.19	0.58	1.00		
SGRQ Impact Score	−0.47	−0.52	−0.43	−0.35	0.71	0.77	1.00	
SGRQ Total Score	−0.45	−0.52	−0.46	−0.31	0.78	0.91	0.95	1.00

Correlation Coefficients in bold were not significant at 95 % confidence

Spearman Rank correlation was used to assess whether there was a relationship between dimensions to ascertain convergent or discriminant validity. Here, similar dimensions (in terms of underlying health construct) correlated whilst unrelated dimensions did not correlate (See Appendix A). For example, the pain dimension in the EQ-5D-3 L and the bodily pain sub-domain within the SF-12 correlated strongly. Additionally, the anxiety dimension in the EQ-5D-3 L and the mental health sub-domain in the SF-12 also correlated strongly. The EQ-5D-3 L self care dimension and the SGRQ symptom score did not correlate. Additionally, both the EQ-5D-3 L dimensions for self-care and usual activity did not correlate with either EQ-5D-3 L dimension for anxiety/depression or the SF-12 dimension of mental health. The EQ-5D-3 L dimension for self-care did not correlate with the SGRQ symptom score either. Lastly, there was no correlation between the role functioning (physical) sub-domain and the mental health sub-domain in the SF-12.

#### Empirical validity

Empirical validity was tested using RE statistics for dichotomised self-reported current general health status and current respiratory health. Tables 6 and 7 shows that that the SF-6D was found to be approximately 56-57 % more efficient than the EQ-5D-3 L at detecting differences in these external measures of health status.

**Table 6:** Empirical validity at 6 months using self-reported general health

Measure at 6 months	Categorisation of self-reported current health	N	Mean	SD	t-statistic	*p*-value	Relative efficiency
EQ-5D-3 L	Very Good or Good	68	0.79	0.23	6.13	P < 0.001	1.57
	Fair, Poor or Very Poor	87	0.50	0.34			
SF-6D	Very Good or Good	67	0.76	0.14	7.69	P < 0.001	
	Fair, Poor or Very Poor	88	0.60	0.12			

N is the sample size. SD denotes the standard deviation. Relative efficiency statistic is referenced at 1.0 for the EQ-5D-3 L. A relative efficiency value higher than 1.0 shows that the SF-6D is more efficient than the EQ-5D-3 L

**Table 7:** Empirical validity at 6 months using self-reported respiratory health

Measure at 6 months	Categorisation of self-reported respiratory health.	N	Mean	SD	t-statistic	*p*-value	Relative efficiency
EQ-5D-3 L	Q1 or Q2	90	0.69	0.31	4.14	*P* < 0.001	1.56
	Q3, Q4 or Q5	33	0.43	0.30			
SF-6D	Q1 or Q2	90	0.70	0.13	5.18	*P* < 0.001	
	Q3, Q4 or Q5	32	0.56	0.10			

N is the sample size. SD denotes the standard deviation. Relative efficiency statistic is referenced at 1.0 for the EQ-5D-3 L. A relative efficiency value higher than 1.0 shows that the SF-6D is more efficient than the EQ-5D-3 L

#### Responsiveness

Table 8 shows the external responsiveness results using self-reported change in current general health as the referent. The change in EQ-5D-3 L utility score ranged from a change of 0.13 for patients who felt that their health was much better to a change of -0.18 for patients who felt that their health was much worse. This mirrored the pattern for the SF-6D where the change in SF-6D utility score ranged from a change of 0.03 for patients who felt that their health was much better to a change of -0.09 for patients who felt that their health was much worse.

**Table 8:** External responsiveness of all PROMs

EQ-5D-3 L	N	6 months	12 months	*P* value	Difference	SRM	SRM CI		Cohen’s D
Much Better	6	0.75	0.88	0.009	0.13	4.09	4.07	4.12	0.85
Better	33	0.67	0.75	0.028	0.08	2.30	2.28	2.31	0.31
Same	79	0.65	0.63	0.448	−0.02	−0.76	−0.77	−0.76	0.06
Worse	31	0.55	0.54	0.686	−0.02	−0.41	−0.42	−0.39	0.05
Much Worse	2	0.69	0.51	0.500	−0.18	−1.00	−1.25	−0.75	0.31
SF-6D	N	6 Months	12 Months	P Value	Difference	SRM	SRM CI		Cohen’s D
Much Better	6	0.71	0.74	0.592	0.03	0.57	0.53	0.62	0.21
Better	37	0.69	0.73	0.014	0.05	2.58	2.58	2.59	0.32
Same	74	0.67	0.69	0.045	0.02	2.04	2.05	2.05	0.15
Worse	28	0.66	0.67	0.814	0.00	0.23	0.23	0.25	0.04
Much Worse	2	0.69	0.60	0.205	−0.09	−3.00	−3.04	−2.96	0.41
SF-12 PCS	N	6 Months	12 Months	P Value	Difference	SRM	SRM CI		Cohen’s D
Much Better	6	35.60	50.40	0.004	14.80	4.95	2.56	7.34	1.36
Better	36	35.07	41.65	0.000	6.58	4.79	4.34	5.24	0.52
Same	71	38.32	40.63	0.002	2.13	2.87	2.70	3.05	0.19
Worse	26	37.45	35.15	0.1517	−2.30	−1.48	−2.08	−0.88	0.18
Much Worse	2	44.01	31.63	0.366	−12.38	−1.54	−12.66	9.57	1.37
SF-12 MCS	N	6 Months	12 Months	P Value	Difference	SRM	SRM CI		Cohen’s D
Much Better	6	49.81	49.39	0.897	−0.42	−0.14	−2.59	2.32	0.03
Better	36	48.15	50.21	0.222	2.06	1.24	−0.70	1.79	0.16
Same	71	44.53	45.24	0.502	0.72	0.68	0.43	0.92	0.05
Worse	26	49.29	46.76	0.226	−2.53	−1.24	−2.02	−0.46	0.20
Much Worse	2	40.00	44.28	0.707	4.28	0.50	−11.46	12.45	0.17
SGRQ Total Score	N	6 Months	12 Months	P Value	Difference	SRM	SRM CI		Cohen’s D
Much Better	4	20.54	13.64	0.012	−6.91	−5.10	−3.77	−6.43	0.54
Better	24	26.50	24.14	0.279	−2.37	−1.11	−0.26	−1.96	0.11
Same	50	30.00	30.53	0.789	0.53	0.27	0.82	−0.28	0.02
Worse	18	36.84	38.14	0.540	2.30	0.63	2.32	−1.07	0.09
Much Worse	1	19.37	54.48	N/A	35.11	N/A	N/A	N/A	N/A

The SF-6D responsiveness results did have an exception where the change in summary scores between the much better (0.03) and better (0.05) self-reported general health categories were in reverse order to that which was expected. The SF-12 PCS summary score ranged from a change of 14.80 for patients who felt that their general health was much better to a change of -12.38 for patients who felt that their general health was much worse. The SF-12 MCS summary score showed much more inconsistency in responsiveness. For patients who felt that their general health was much better, the SF-12 MCS summary score produced a change of -0.42 which indicated that mental health had decreased over time. A similar phenomenon was also seen for the self-reported category of “much worse” general health, where patients who felt much worse produced a change in the SF-12 MCS summary score of 4.28 indicating that although patients felt much worse, mental health had improved. Finally the SGRQ total score (0–100 with a higher score representing a lower respiratory health) ranged from a change of -6.9050 for patients who felt that their health was much better to a change of 35.1100 for patients who felt that their health had deteriorated. The SGRQ responsiveness followed the general trend that was estimated. Effect sizes were consistently ordered among the EQ-5D-3 L and SGRQ with the EQ-5D-3 L generating larger effect sizes compared to the SGRQ. Effect sizes for the SF-12 and its SF-6D derivative were smaller.

## Discussion

The aim of this study was to compare and assess the psychometric properties of the EQ-5D-3 L, SF-12 and its preference based derivative the SF-6D, and the SGRQ, in patients with moderate to severe ARDS. This study aimed to provide evidence for the use of generic and condition specific PROMs in future clinical trials and trial based economic evaluations associated with critical care and specifically ARDS. The results of the study showed significant variation between properties. Response rates were varied with the EQ-5D generating the highest response rates and the SGRQ generating the lowest response rates. Cronbach’s alpha scores showed that all PROMs were deemed acceptable to the study population. Results also showed that there was no statistically significant difference between known groups with minimal effect sizes. All utility and summary scores correlated statistically with the exception of the SF-12 PCS and MCS scores which did not correlate. When assessing the empirical validity of the EQ-5D-3 L compared to the SF-6D, results showed that the SF-6D was an efficient and empirically valid alternative to the EQ-5D-3 L. Lastly, the EQ-5D-3 L and SGRQ were more responsive compared to the SF-12 and SF-6D with the EQ-5D-3 L generating greater effect sizes than the SGRQ.

When comparing and assessing the psychometric properties of PROMs, there are specific difficulties that need to be addressed to provide transparency of the analysis. For instance, HRQoL can be measured in different ways: the EQ-5D-3 L and SF-12 are generic PROMs and measure general health whereas the SGRQ is a condition specific PROM that measures HRQoL in relation to respiratory concerns. Each of these PROMs also measures HRQoL over different time periods: the EQ-5D-3 L asks individuals about their health state “today,” whilst the SF-12 and the SGRQ measures utilised in the OSCAR study ask individuals about their health during or over the past 4 weeks.

High response rates were seen for all measures due, in part, to the strategy adopted to maximise patient responses. A letter was sent out to survivors 2 weeks before the follow up questionnaires were due at 6 and 12 months, as a reminder to the patient that they would be receiving a questionnaire related to their health from the trial group in the next few weeks. When the follow up questionnaire was sent to patients, a freepost envelope was also sent to maximise response rates. The PROMs within the follow up questionnaires were ordered as follows: the SGRQ, the EQ-5D-3 L and then the SF-12. This may partly explain our response rates where the highest response rate was observed for the SGRQ symptom score. Hence, a large symptom score response rate may be due to the patient having to first answer the first part of the SGRQ (Questions 1–8), which results in a symptom score. The second part of the SGRQ (Questions 9–16) results in activity and impact scores which had lower response rates. Therefore, due to the volume of the SGRQ (50 items), participant fatigue and potential repeatability of dimensions relating to respiratory health may have led to lower response rates for the latter half of the SGRQ.

The EQ-5D-3 L was found to have an acceptable reliability and internal consistency. The SF-6D and SF-12 showed greater reliability and internal consistency than the EQ-5D-3 L. The SGRQ had high Cronbach’s alpha statistics that exceeded 0.95. Scores higher than 0.95 are not necessarily desirable, as this indicates that the items may be entirely redundant [45]. Hence, there is a possibility of item reduction or creating a derivative within the SGRQ that could potentially aid in increasing response rates, although this has to be counter-balanced against the broader goals of the measure.

Construct validity was tested using known group’s comparisons. With no outcomes measures being collected at baseline, the known groups comparison was a partial analysis to ascertain construct validity. For the known groups based on APACHE II scores and the PaO_2_/FiO_2_ ratio, it was found that there was no difference between known groups and all effect sizes were small. This highlights the need for research to be conducted on measuring and valuing health for the unconscious health state in ARDS patients so that baseline assessments can also be conducted.

There was no correlation between SF-12 PCS and MCS scores which would be expected as they are delineated across different dimensions, which should provide divergent constructs that are not overlapping. There was strong correlation between the EQ-5D-3 L and the SF-6D. All four SGRQ summary scores correlated highly with each other, further highlighting the overlap between its dimensions and constructs. This could be due to the SGRQ being a condition specific measure but also due to the greater amount of items used within the SGRQ. The majority of Spearman Rank correlations were statistically significant at the 95 % confidence level, which shows that there was overlapping constructs between measures. There was particularly strong correlation between the pain dimension in the EQ-5D-3 L and bodily pain sub domain in the SF-12. There was also a strong correlation between the anxiety dimension in the EQ-5D-3 L and the mental health sub-domain in the SF-12. Hence, both results provide evidence for convergent validity. Furthermore, EQ-5D self care and EQ-5D usual activities did not correlate with the anxiety/depression dimension of the EQ-5D-3 L or the mental health sub-domain of the SF-12 which shows some evidence for discriminant validity between dimensions or domains. All in all, correlation at both the utility/summary score level and dimension specific level generated values which revealed expected overlapping and non-overlapping constructs. Hence, the PROMs displayed convergent and discriminant validity.

Empirical validity was tested using relative efficiency statistics to see whether the EQ-5D-3 L and SF-6D generated utility or summary scores that reflected hypothesised differences in external indicators of health status for current general health and total respiratory health. For both the external indicators of current general health and total respiratory health, the SF-6D was found to be more efficient than the EQ-5D and therefore considered to be an empirically valid alternative multi attribute utility measure to the EQ-5D. This shows that the SF-6D is capable of discriminating between external indicators of health status in keeping with the results of other studies [39].

As no baseline outcome measures were collected, it was difficult to comprehensively test internal responsiveness and external responsiveness and hence basic floor and ceiling effects were used to assess responsiveness. The EQ-5D-3 L generated ceiling effects where respondents chose the highest response on ordinal scales that cannot be improved. In order to address this issue, the EQ-5D-5 L has been created where there are five response levels within each dimension, which should decrease ceiling effects [46]. The SF-12 and SF-6D had an advantage in responsiveness due to lack of or absence of floor or ceiling effects. The SGRQ had large ceiling effects, which shows that it has a limitation in its ability to register health changes.

External responsiveness was also analysed where a reference measure, Question 1 of the SF-12 that focuses on current general health, was used to assess whether the changes registered by a measure over time resemble those expected based on an external measure of health. This reference measure was chosen as it is not used in the calculation of any utility or summary scores. We found that the EQ-5D-3 L and SGRQ were more responsive than the SF-12 and SF-6D. As a small sample has been analysed not all mean comparisons are statistically significant or could be performed. Additionally, there may be a response shift bias, which indicates that a patient’s values for health changed over the course of time in the SF-6D, which is further driven by the SF-12 PCS Score.

Many of the differences between the EQ-5D-3 L, SF-6D and SGRQ were expected considering differences in their descriptive system, scoring function, valuation and range of utility and scoring systems. Furthermore, poor health states are valued more highly (in utility terms) in the SF-6D compared to the EQ-5D-3 L [25, 47, 48]. Additionally, the SF-6D is generally better able to detect smaller changes in health compared to the EQ-5D-3 L [48]. There is also evidence which suggests that the time trade off method results in greater values for mild or moderate health states and lower values for severe health states, which may partly explain our findings [47].

The limitations of this study include having a small sample of responders which limited some analyses and decreasing the generalizability of our results. This study was also disadvantaged as no data regarding HRQoL was collected at baseline. Instead, patients were assumed to have an EQ-5D-3 L utility score of -0.402 (representing an unconscious health state) in the separate trial-based economic evaluation [49]. Unconsciousness is not a defined health state by the SF-12 or the SGRQ and hence there were no pre-defined SF-12 or SGRQ QoL or HRQoL values for the baseline unconscious health state. In the current EQ-5D-3 L value set, the unconscious state has been assigned a utility value of -0.402 [49]. This value suggests that the general public, on average, considers unconsciousness to be “worse than dead (<0)” but better than being conscious and experiencing problems on all dimensions (-0.543). Future research must clearly take into account methodological issues surrounding measuring and valuing the unconscious health state in critical care. Issues include whether being unconscious in this setting represents one health state or a number of health states, if an individual has any feelings or emotions when unconscious, whether people can value unconsciousness without knowledge of the preceding and subsequent health states and whether being asleep is equivalent to being unconscious for a short time.

Lastly, in order to determine the robustness of the EQ-5D-3 L and SF-6D utility scores in this population, advocacy for mapping research is encouraged. Mapping exercises could be based on using the SGRQ summary scores from OSCAR or similar trials and mapping onto the EQ-5D-3 L, EQ-5D-5 L and SF-6D in order to assess if there is any difference in the original utility scores derived from the OSCAR trial and the estimated utility scores derived from the mapping exercises.

## Conclusion

This study highlights the complications that can arise when trying to assess the psychometrics of PROMs in intensive care contexts and hence advocates for researchers and policy makers to notice this gap in evidence and follow through with building evidence surrounding utility values for the unconscious health state. In summary, it was considered that generic instruments were suitable to measure HRQoL in this population and showed good properties for most criteria whereas more consideration has to be given to the role of condition specific instruments in this context. The selection of a preferred PROM for evaluative studies within the ARDS context should ultimately depend on the relative importance placed on individual psychometric properties and the importance placed on generation of health utilities for economic evaluation purposes.

**Table 9:** Correlation between dimensions

	EQ5D Mobility	EQ5D Self care	EQ5D Usual activity	EQ5D Pain	EQ5D Anxiety depression	SF12 General health	SF12 Physical Functioning	SF12 Role functioning (Physical)	SF12 Role functioning (Emotional)	SF12 Bodily pain	SF12 Mental health	SF12 Vitality	SF12 Social functioning	SGRQ Symptom score	SGRQ Activity score	SGRQ Impact score	SGRQ Total score
EQ5D Mobility	1.00																
EQ5D SelfCare	0.47	1.00															
EQ5D UsualActivity	0.56	0.40	1.00														
EQ5D Pain	0.48	0.60	0.36	1.00													
EQ5D Anxiety Depression	0.23	**0.14**	**0.15**	0.33	1.00												
SF12 General Health	−0.57	−0.45	−0.49	−0.53	**−0.19**	1.00											
SF12 Physical Functioning	−0.63	−0.57	−0.68	−0.62	−0.24	0.64	1.00										
SF12 Role Functioning (Physical)	−0.51	−0.54	−0.68	−0.62	−0.23	0.63	0.77	1.00									
SF12 Role Functioning (Emotional)	−0.33	−0.30	−0.32	−0.42	−0.79	0.33	0.36	0.42	1.00								
SF12 Bodily Pain	−0.52	−0.59	−0.32	−0.81	−0.28	0.55	0.65	0.63	0.33	1.00							
SF12 Mental Health	−0.27	**−0.16**	**−0.05**	−0.31	−0.80	0.25	0.25	**0.21**	0.68	0.34	1.00						
SF12 Vitality	−0.35	−0.24	−0.31	−0.28	−0.32	0.45	0.46	0.37	0.34	0.34	0.45	1.00					
SF12 Social Functioning	−0.31	−0.38	−0.32	−0.43	−0.63	0.39	0.45	0.42	0.68	0.45	0.61	0.36	1.00				
SGRQ Symptom Score	0.25	**0.12**	0.29	0.23	0.30	−0.46	−0.42	−0.30	−0.26	−0.28	−0.31	−0.36	−0.33	1.00			
SGRQ Activity Score	0.43	0.24	0.48	0.33	−0.25	−0.48	−0.58	0.44	−0.30	−0.28	−0.22	−0.41	−0.22	0.61	1.00		
SGRQ Impact Score	0.32	0.27	0.45	0.38	0.33	−0.45	−0.59	−0.46	−0.36	−0.29	−0.30	−0.45	−0.32	0.69	0.85	1.00	
SGRQ Total Score	0.38	0.26	0.45	0.36	0.30	−0.49	−0.59	−0.44	−0.33	−0.30	−0.28	−0.45	−0.29	0.77	0.94	0.96	1.00

Values in bold did not correlate at 95 % confidence

## Abbreviations

APACHE II: acute physiology and chronic health evaluation
ARDS: acute respiratory distress syndrome
CI: confidence interval
EQ-5D-3 L: euroqol 5 dimensions 3 levels
EQ-5D-5 L: euroQol 5 dimensions 5 levels
H_2_O: water
HFOV: high frequency oscillatory ventilation
HRQoL: health-related quality of life
ICU: intensive care unity
MCS: mental component score
ONS: office of national statistics
OSCAR: oscillation in ARDS study
PCS: physical component score
PEEP: positive end expiratory pressure
PROMs: patient reported outcome measures
PROs: patient reported outcomes
Q1, Q2, Q3, Q4, Q5: quintile X
QALY: quality adjusted life year
QoL: quality of life
RE: relative efficiency
SD: standard deviation
SF-12: short form 12
SF-6D: short form 6D
SG: standard gamble
SGRQ: St Georges respiratory questionnaire
TTO: time trade off method
VAS: visual analogue scale
